# Detection of Recombinant Rousettus Bat Coronavirus GCCDC1 in Lesser Dawn Bats (*Eonycteris spelaea*) in Singapore

**DOI:** 10.3390/v12050539

**Published:** 2020-05-14

**Authors:** Adrian C. Paskey, Justin H. J. Ng, Gregory K. Rice, Wan Ni Chia, Casandra W. Philipson, Randy J.H. Foo, Regina Z. Cer, Kyle A. Long, Matthew R. Lueder, Xiao Fang Lim, Kenneth G. Frey, Theron Hamilton, Danielle E. Anderson, Eric D. Laing, Ian H. Mendenhall, Gavin J. Smith, Lin-Fa Wang, Kimberly A. Bishop-Lilly

**Affiliations:** 1 Department of Microbiology and Immunology, Uniformed Services University of the Health Sciences, Bethesda, MD 20814 USA; adrian.paskey@usuhs.edu (A.C.P.); eric.laing@usuhs.edu (E.D.L.); 2 Genomics and Bioinformatics Department, Biological Defense Research Directorate, Naval Medical Research Center–Frederick, Fort Detrick, MD 21702 USA; gregory.k.rice.ctr@mail.mil (G.K.R.); casandra.philipson@ll.mit.edu (C.W.P.); regina.z.cer.ctr@mail.mil (R.Z.C.); kyle.a.long8.ctr@mail.mil (K.A.L.); matthew.r.lueder.ctr@mail.mil (M.R.L.); kenneth.g.frey4.civ@mail.mil (K.G.F.); theron.hamilton.mil@mail.mil (T.H.); 3 Leidos, Reston, VA 20190, USA; 4 Programme in Emerging Infectious Diseases, Duke-NUS Medical School, Singapore 169857, Singapore; justin.ng.hj@gmail.com (J.H.J.N.); wanni.chia@duke-nus.edu.sg (W.N.C.); randy.foo@duke-nus.edu.sg (R.J.H.F.); xiaofang.lim@duke-nus.edu.sg (X.F.L.); danielle.anderson@duke-nus.edu.sg (D.E.A.); ian.mendenhall@duke-nus.edu.sg (I.H.M.); gavin.smith@duke-nus.edu.sg (G.J.S.); linfa.wang@duke-nus.edu.sg (L.-F.W.); 5 Defense Threat Reduction Agency, Fort Belvoir, VA 22060 USA

**Keywords:** lesser dawn bat, Rousettus bat coronavirus GCCDC1, recombinant, coronavirus

## Abstract

Rousettus bat coronavirus GCCDC1 (RoBat-CoV GCCDC1) is a cross-family recombinant coronavirus that has previously only been reported in wild-caught bats in Yúnnan, China. We report the persistence of a related strain in a captive colony of lesser dawn bats captured in Singapore. Genomic evidence of the virus was detected using targeted enrichment sequencing, and further investigated using deeper, unbiased high throughput sequencing. RoBat-CoV GCCDC1 Singapore shared 96.52% similarity with RoBat-CoV GCCDC1 356 (NC_030886) at the nucleotide level, and had a high prevalence in the captive bat colony. It was detected at five out of six sampling time points across the course of 18 months. A partial segment 1 from an ancestral Pteropine orthoreovirus, p10, makes up the recombinant portion of the virus, which shares high similarity with previously reported RoBat-CoV GCCDC1 strains that were detected in Yúnnan, China. RoBat-CoV GCCDC1 is an intriguing, cross-family recombinant virus, with a geographical range that expands farther than was previously known. The discovery of RoBat-CoV GCCDC1 in Singapore indicates that this recombinant coronavirus exists in a broad geographical range, and can persist in bat colonies long-term.

## 1. Introduction

Human coronaviruses (HCoVs) have been studied for just over half a century, and are a priority for research as a consequence of recent high-profile disease outbreaks [1,2]. The first coronavirus detection from bats was reported in 2005, following increased surveillance of wildlife in response to the severe acute respiratory syndrome coronavirus (SARS-CoV) outbreak [3]. Since then, 3796 coronaviruseshave been detected in feces, swabs, tissue, blood, and urine of wild collected bats from surveillance research spanning 58 countries (Database of Bat-Associated Viruses as of November 2019) [4]. Of the seven known human coronaviruses, four are regarded as causes of mild to self-limiting disease, and circulate endemically: HCoV-NL63, HCoV-229E, HCoV-OC43 and HKU1 [5]. Of the remaining HCoVs, SARS-CoV and Middle Eastern respiratory syndrome coronavirus (MERS-CoV) are WHO R&D Blueprint priority pathogens, that represent potential epidemic threats lacking effective on-hand countermeasures [2,6]. The most recently discovered human coronavirus and causative agent of the COVID pandemic, SARS-CoV-2, was first detected in December 2019 in Wuhan, China, is known to cause mild to severe respiratory disease in humans, and is most similar to bat SARS-like CoVs on a sister clade to SARS-CoV [7]. SARS-CoV and MERS-CoV are transmitted to humans through palm civets and camels, respectively, where the terrestrial mammals act as amplifying intermediate hosts [8]. Phylogenetic reconstructions suggest that the putative ancestors of both viruses are bat coronaviruses [9,10,11]. Although members of the orthocoronavirinae genera have been found to infect a wide diversity of animal hosts, alpha- and betacoronaviruses are thought to have bat origins, whereas gamma- and deltacoronaviruses are thought to be of avian origin [12,13]. 

The viral family *Coronaviridae* is so named due to the morphology of the virion, which resembles a crown; thus, the name is derived from the Greek word for crown [12]. Coronaviruses are enveloped positive-sense and single-stranded RNA viruses, with non-segmented genomes approximately 30 kb in length [14]. Subgenomic mRNA generated during viral replication increases the likelihood for homologous recombination with coinfecting viruses by way of template switching, resulting in novel variants [15]. Coronavirus genomes undergo a high frequency of recombination, particularly in the spike gene, which expresses the envelope spike protein responsible for viral entry into host cells [16]. This potentially leads to an increase in the capacity to infect a wider range of hosts and/or altered virulence [16]. Cross-family recombination of viruses is unusual, and can bring rise to the emergence of distinct mammalian or plant viruses. For example, recombination between caliciviruses and retroviruses, which belong to different virus families but infect the same avian host, has been implicated in the generation of novel avian and porcine circoviruses [17]. Viral recombinants that emerge from animal reservoirs may have the potential to infect a broader spectrum of intermediate hosts, or spill over into human populations. For this reason, recombination of potentially zoonotic viruses has been studied extensively, with the intent to predict the emergence of virulent, recombinant coronaviruses [16].

SARS-CoV, SARS-CoV-2, MERS-CoV and Rousettus bat coronavirus HKU9 (RoBat-CoV HKU9) are ancestrally related as bat-borne betacoronaviruses [7]. RoBat-CoV HKU9 was first reported in 2007, and is one of numerous betacoronaviruses discovered during biosurveillance sampling in the wake of the SARS-CoV outbreak to identify the animal source [18]. In addition to hosting coronaviruses, bats are regarded as the primary animal hosts of an exceptionally diverse level of viruses, including paramyxoviruses (e.g., Nipah virus), filoviruses (e.g., Marburg virus) and orthoreoviruses [19,20,21,22]. Orthoreoviruses are non-enveloped viruses with 10 genome segments comprised of double-stranded RNA. Multiple fusogenic orthoreoviruses, defined by their ability to cause cell syncytia, circulate in Southeast Asian bats and are known to cause disease in humans [23]. As animal reservoirs for diverse families of viruses, bats represent a mammalian host in which co-infecting viruses could potentially recombine [24]. A cross-family recombinant virus was discovered in a wild population of Leschenault’s rousette (*Rousettus leschenaultii*) bats in 2016 in China [25]. This virus, Rousettus bat coronavirus GCCDC1 (RoBat-CoV GCCDC1), possesses a partial segment 1 from Pteropine orthoreovirus incorporated into the backbone of Rousettus bat coronavirus HKU9 between the N and NS7a genes.

Following the discovery of RoBat-CoV GCCDC1 in Leschenault’s rousette in 2016, in Yúnnan, China, this virus was further detected in a lesser dawn bat (*Eonycteris spelaea*) population known to co-roost with Leschenault’s rousette [26]. It is thought that the dense population of bat roosts and gregarious behavior contribute to the persistence of RoBat-CoV GCCDC1 [27]. In an analysis of viral families, coronaviruses were found to have a level of topological distance between phylogenetic trees from known hosts, that suggests frequent cross-species transmission among mammals and host switching [28]. In this study, we conducted longitudinal virome analysis of a wild-caught, captive colony of lesser dawn bats in Singapore. We recently reported the virome analysis of this colony [29], which was established from a wild colony known to host a lineage D betacoronavirus [30]. Using a hybridization-based targeted enrichment sequencing approach, and sequencing using the MiSeq platform [31], we detected genomic evidence of RoBat-CoV GCCDC1 in this colony of lesser dawn bats, and then performed unbiased shotgun sequencing using the NextSeq 500 platform. This is now the third report of this recombined coronavirus/orthoreovirus in a bat host, and the first report of the persistence of this virus in bats beyond the geographical region of Yúnnan, China. In the current study, we investigate colony-level persistence and genetic conservation of this recombinant virus in captive lesser dawn bats. The findings of this study are of significant relevance to Asia-Pacific regional public health laboratories, due to the implications for disease prevention and control.

## 2. Materials and Methods 

### 2.1. Vertebrate Animal Care and Safety

All bats were housed and handled at Duke-National University of Singapore Medical School and National Large Animal Research Facility (NLARF) animal facilities. Trained laboratory personnel provided daily care for the animals according to the guidelines agreed upon by Duke-NUS Institutional Animal Care and Use Committee (2015/SHS/1088) and the Agri-Food and Veterinary Authority of Singapore. All sampling was noninvasive. 

### 2.2. Bat Colony Structure and Sampling Strategy

Sampling was performed as previously described [29]. In summary, bats were housed in stainless steel mesh cages with ample room for roosting, and swabs were collected quarterly for health screening purposes. Age at the time of capture from the wild was unknown, and bats were excluded from sampling while juveniles (if born into the colony during the study) or pregnant. Head, body, oral and rectal swabs were obtained using sterile polyester tipped swabs and stored in 2 mL screw cap micro tubes (Sarstedt, Germany) containing 500 µL viral transport media (VTM, 10% Bovine Serum Albumin, 20% Antibiotics-Antimycotic in milli-Q water) at −80 °C. 

### 2.3. Nucleic Acid Extraction and Sequencing

Extractions and sequencing were performed as previously described [29]. Briefly, RNA was extracted from head, body, oral and rectal swabs of each bat using a QIAGEN RNeasy Kit with on-column DNase digestion (Qiagen; Valencia, CA). RNA was eluted twice with RNase-free water. Conventional high throughput sequencing (HTS; shotgun) libraries were multiplexed for sequencing on the NextSeq500 platform using v2 chemistry with 2 × 150 bp read lengths. Post-library enrichment probe targets and preparation methods were previously described by Paskey et al.; samples were probed in pools of 12 and multiplexed for sequencing on the MiSeq platform using v3 chemistry with 2 × 300 bp read lengths [31]. 

### 2.4. Bioinformatic Analyses

Rousettus bat coronavirus GCCDC1 was first detected in target-enrichment samples by read-mapping to Rousettus bat coronavirus isolate GCCDC1 356 (NC_030886) using CLC Genomics Workbench V11 (QIAGEN Bioinformatics; Redwood City, CA). Further analysis for prevalence of the related strain was performed using shotgun data mapped using bbsplit with parameter adjustment of minid = 0.75 [32]. Results were filtered to require both forward and reverse reads covering more than 100 bases and at least 10 reads (minimum of 95% identity). Multiple sequence alignments, as well as variant analysis of contigs and reads, were performed with CLC Genomics Workbench V11 (QIAGEN Bioinformatics; Redwood City, CA). Dinucleotide variation was evaluated by calculating Rho using seqinr package for R [33,34]. Shotgun reads for the completely assembled sequence RoBat-CoV Singapore (GenBank accession number MT350598) were from a single head swab collected from bat 7634D82 in October 2016 (raw reads available in BioProject PRJNA561193), using 1,485 reads with an average coverage depth of 7.12x.

### 2.5. Phylogenetic Analysis

Molecular phylogenetic analysis was performed with 100 bootstrap replicates, using the Maximum Likelihood method based on the General Time Reversible model in MEGA7 [35]. The tree with the highest log likelihood (–74447.85) is shown. The analysis involved four nucleotide sequences: the genome sequence generated from this study (RoBat-CoV GCCDC1 Singapore), GCCDC1 356 (KU762338), GCCDC1 346 (KU762337) and HKU9-1 (NC_009021). All positions containing gaps and missing data were eliminated. There were a total of 28,712 positions in the final dataset. 

### 2.6. Geographic Range of Bats

Leschenault’s rousette and lesser dawn bat range data were obtained in shapefiles format from the International Union for Conservation of Nature and Natural Resources (ICUN) [36]. Ranges were mapped using ‘tmap’ in R [34,37].

## 3. Results

### 3.1. Prevalence of GCCDC1 in the Captive Colony

Genomic evidence of RoBat-CoV GCCDC1 was detected in 72 of 206 samples that were collected from a captive colony of lesser dawn bats in 2016 and 2017, and characterized via shotgun sequencing. The virus was detected in five of six sampling dates in head, body, oral and rectal swabs (Table 1), and this included mucosal sites (oral and rectal swabs) in four of five time points (Supplemental Table S1). Previous longitudinal studies that evaluated the persistence of RoBat-CoV GCCDC1 in wild bats in Yúnnan province, China, reported a prevalence of 39.3% in 2014, 35.6% in 2015 [27], as well as another report of 5.26% in 2015 and 18.87% in 2016 [26]. The average prevalence among sequenced swabs in this study of captive bats was 38.1% in 2016 and 3.6% in 2017. 

### 3.2. Comparison to Previously Discovered Cross-Family Recombinant Coronaviruses

**Figure 1:**
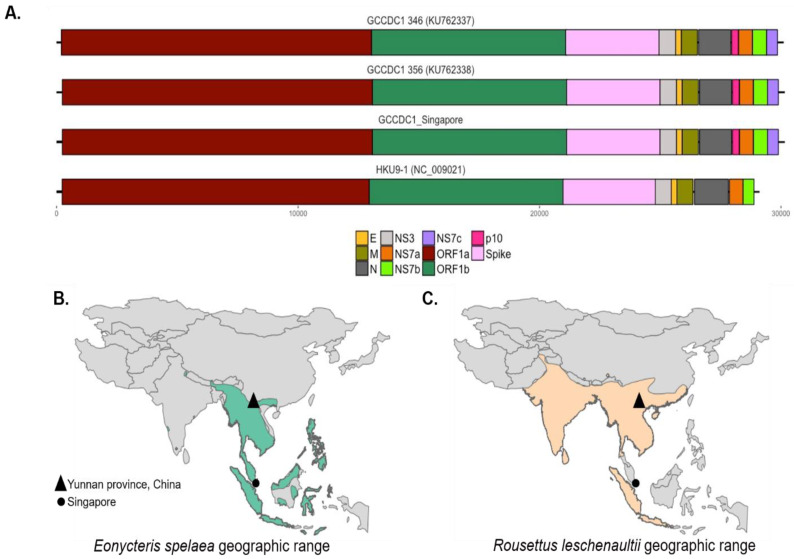
RoBat-CoV GCCDC1 Singapore is genetically similar to the strain detected in China and its host is found in a wide geographic range. (**A**) The distribution of orthoreovirus p10 gene across three strains of RoBat-CoV GCCDC1—346, 356 and Singapore—as well as a putative parental relative, RoBat-CoV HKU9-1. Note the presence of the p10 gene insertion (dark pink) and accessory gene NS7c (purple) only in the RoBat-CoV GCCDC1 strains. The geographic distribution of (**B**) *E. spelaea* and (**C**) *R. leschenaultii* across Southeast Asia, respectively [36].

The genome of RoBat-CoV GCCDC1 includes a p10 gene from segment S1 of an ancestral orthoreovirus, inserted between the RoBat-CoV HKU9 nucleocapsid (N) and NS7a accessory genes (Figure 1A). The p10 gene encodes a fusion-associated small transmembrane (FAST) protein that surface-localizes and causes cell-to-cell syncytia, as shown by Huang et al. [25]. The same transcription regulatory sequence (TRS) motif, 5′-ACGAAC-3′, is shared between RoBat-CoV GCCDC1 and RoBat-CoV HKU9, with the identical alteration of one nucleotide to ‘TCGAAC’ in the intergenic TRS before the envelope gene in both RoBat-CoV GCCDC1 and HKU9 [18,25]. Previously reported RoBat-CoV GCCDC1 sequences detected in China share a high identity [26]. The nucleotide similarity between the full genome detected in Singapore and RoBat-CoV GCCDC1 356 is 96.52%. We report notable similarity between strains despite host and geographic differences. RoBat-CoV GCCDC1 was first detected in China where the geographic range of Leschenault’s rousettes and lesser dawn bats overlap (triangles, Figure 1B–C).

**Figure 2:**
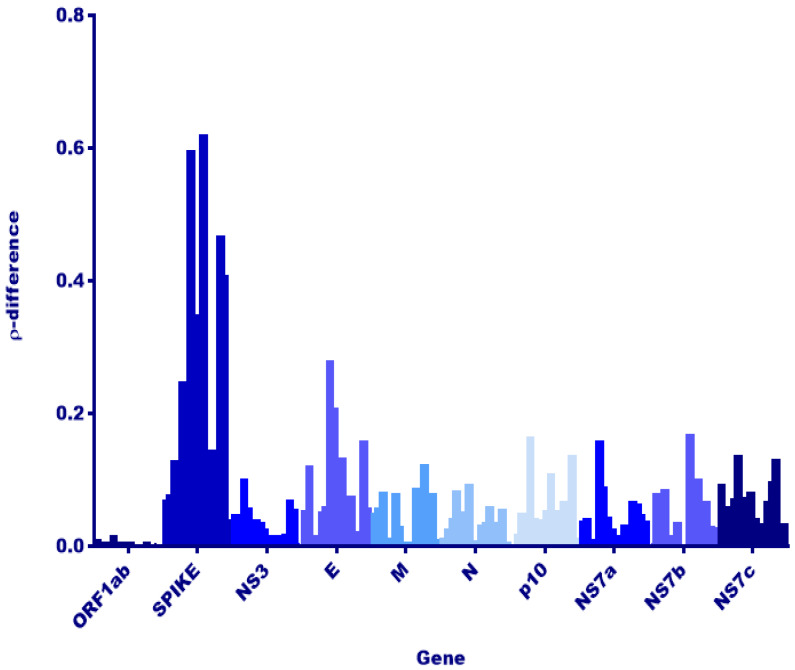
Dinucleotide usage analysis among strains RoBat-CoV GCCDC1 356 and RoBat-CoV GCCDC1 Singapore. Rho-difference values (ρ-difference, y-axis) were calculated, using the results from dinucleotide analysis of each gene (genes shown in shades of blue, x-axis), to evaluate any significant differences among RoBat-CoV GCCDC1 strains detected in different hosts (*E. spelaea* and *R. leschenaultii* species). By two-way analysis of variance (ANOVA), the rho-difference for only the spike gene is significantly different from all other genes (alpha 0.05, *p* < 0.0001), and is a possible indication of differences due to replication within distinct hosts.

**Figure 3:**
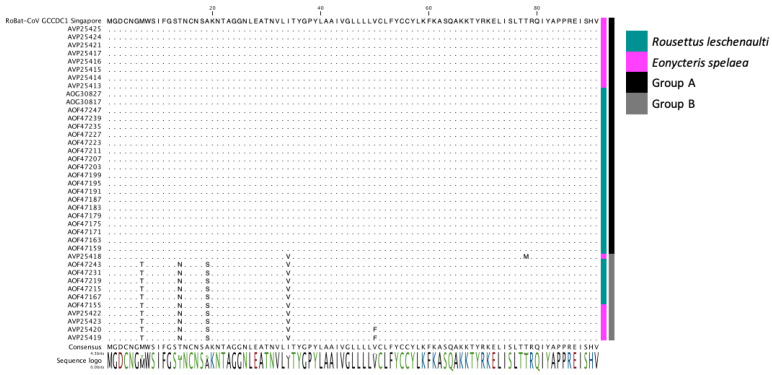
Multiple sequence alignment (MSA) of the amino acid sequence for p10 from RoBat-CoV GCCDC1 of the strain detected in this study, and p10 sequences reported from Yúnnan province, China [25,26,27]. The colored bars to the right of the MSA indicate that the sequence was detected in *R. leschenaultii* (dark blue) or *E. spelaea* (pink). Groups A (black) and B (grey) were previously defined in the literature [27].

Next, we investigated viral genes that would reflect host adaptation or interspecies codon usage bias [38,39]. Dinucleotide analysis of each gene and rho value calculation was utilized to evaluate any host replication biases reflected among *E. spelaea* and *R. leschenaultii* species. A dinucleotide bias among strains of RoBat-CoV GCCDC1 has not been previously investigated, and we hypothesized that such a bias, if detected, may result from circulation in distinct bat host species. Upon investigation, we detected no significant difference in rho value or notable variation at the nucleotide level in the reovirus-derived segment p10 (Figure 2). Furthermore, we detected a high level of conservation for p10 at the amino acid level between *E. spelaea* (Singapore), *E. spelaea* (China) and *R. leschenaultii* RoBat-CoV GCCDC1 (Figure 3). The p10 amino acid sequence of the Singapore strain is 98.6% similar at the nucleotide level to previously published strains 346 and 356, falling within the ‘Group A’ categorization of p10 sequences described by Obameso et al. [27].

### 3.3. Detection of the Recombinant Genome despite Geographic and Host Differences

**Figure 4:**
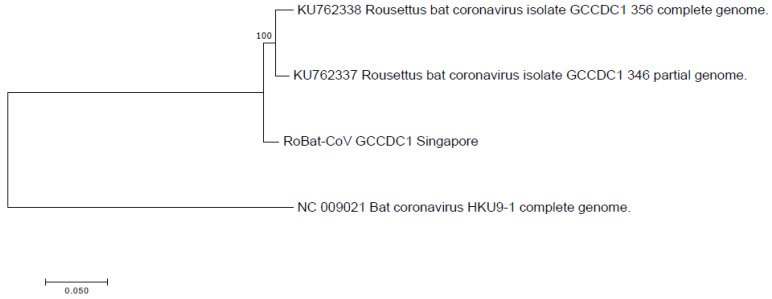
Phylogenetic analysis of RoBat-CoV GCCDC1 Singapore strain as compared to previously published references, and related RoBat-CoV HKU9. Nucleotide-level analysis was performed using full genome alignments and the maximum likelihood method, based on the General Time Reversible model in MEGA7 with 100 bootstrap replicates [35].

We did not detect the putative parental strain (RoBat-CoV HKU9) of this recombinant in the sampled cohort of captive lesser dawn bats. Moreover, the p10 segment of RoBat-CoV GCCDC1 Singapore is genetically distinct from known orthoreoviruses. Given that the necessary parental strains were not detected in the captive colony, we extrapolate from our observations that the recombinant is circulating in wild lesser dawn bats, and did not recently arise as a recombinant in the captive colony. Furthermore, we observe that the backbone of known RoBat-CoV GCCDC1 strains are closely related, and distinct at the nucleotide level from RoBat-CoV HKU9. The phylogenetic analysis of the full nucleotide sequence, as compared to previously published references for strains of GCCDC1 and RoBat-CoV HKU9 (Figure 4), illustrates the relatedness of RoBat-CoV GCCDC1 sequences. The paired reads and contigs that span both junctional regions of p10 provides evidence that the sequence data represent a true recombinant virus. Furthermore, we detected the shedding of RoBat-CoV GCCDC1 using both NextSeq 500 and MiSeq platforms, by shotgun and targeted enrichment sequencing, respectively.

## 4. Discussion

The evolution of viruses of pandemic potential, such as bat-borne coronaviruses, is significant to public health due to the risk of spillover and subsequent sustained human-to-human transmission. Viral recombination in host reservoirs is a concern for public health, as these events can increase the potential for spillover. Here, we report genomic evidence of the recombinant RoBat-CoV GCCDC1 in a population of lesser dawn bats in Singapore. Interestingly, this recombinant virus strain exhibits genetic conservation, as compared to strains initially detected in Yúnnan, China. Therefore, we have demonstrated that a cross-family recombinant coronavirus persists in a captive colony of bats, and is similar at the nucleotide level to previously discovered strains, despite geographic and host differences. There is high similarity between RoBat-CoV GCCDC1 Singapore and previously reported strains by gene arrangement (Figure 1A), and high conservation at the amino acid level within the p10 insertion (Figure 2), and across the whole genome at the nucleotide level (Figure 3). It is not surprising that the spike gene is the region with the greatest dinucleotide variation among strains (Figure 2). This may indicate that, while RoBat-CoV GCCDC1 356 and Singapore may be overall highly related, the spike protein is under increased selection pressure compared to the rest of the genome. One unusual element of the backbone of RoBat-CoV GCCDC1 is the presence of nonstructural protein-encoding accessory gene NS7c at the 3′ end of the genome. This genome arrangement is most similar to that of deltacoronaviruses, which can infect humans, but are typically found in birds or pigs [40,41]. The betacoronavirus related to HKU9-1 is found in bats and does not possess NS7c [42]. Taken together, it is possible that RoBat-CoV GCCDC1 is the product of several historical recombination events.

The virus was detected less frequently in the second year of the longitudinal study. It is unclear if prevalence of RoBat-CoV GCCDC1 was impacted during the first year of the study by unknown confounding factors. Development of a serological assay for RoBat-COV GCCDC1, paired with qPCR and/or high throughput sequencing (HTS), could expand our ability to understand the viral dynamics within bat colonies in the future. We hypothesize that RoBat-CoV GCCDC1 was able to persist in this lesser dawn bat colony, but, with no immigration or emigration, it was not continually shed at levels that were detectable by our methods. This work indicates the ability for the virus to persist long-term within a captive colony of lesser dawn bats. It is important to note that this study evaluated the population persistence of RoBat-CoV GCCDC1 in a captive colony, which is unique from previous longitudinal reports in wild-caught bats, which are exposed to variables such as weather changes or the ability to exchange genetic viral variants via dispersal and migration. The geographic distance and genetic similarity between strains provide insight to the possibility that this strain likely exists in the geographical region between Yúnnan province and Singapore. Additionally, unbiased biosurveillance assays could detect other, yet-to-be-discovered cross-family recombinants.

Evidence of coronavirus infection has been detected in 198 bat species by a variety of methods, such as conventional polymerase chain reaction (PCR), quantitative PCR, serology, and HTS [4]. Coronaviruses are of high priority for biosurveillance, due to their propensity to evolve quickly, prior history of zoonotic spillover, and subsequent high mortality rates in humans [16]. Surveillance for coronaviruses of pandemic potential often targets the polymerase gene by screening noninvasive samples via PCR assays. Immunoassays are also invaluable in detecting previous exposure of viruses of zoonotic potential in reservoirs, but they do not provide information with regard to current virus infection [43]. One shortcoming of these approaches is the inherent bias toward known viruses, specifically highly conserved genomic regions, which can be circumvented with HTS. Progenitor coronavirus strains related to SARS-CoV or SARS-CoV-2 may exist in a cohort of diverse viral variants in reservoirs [24], and this depth of information can be discovered via an unbiased approach like HTS. Genetic diversity of viruses within a single host reservoir may permit frequent transmission to incidental hosts, which becomes problematic when a genetic variant capable of infecting the new host population spills over [44]. Given the propensity of coronaviruses to recombine, unbiased HTS, as used in this study, provides insight to the diversity of coronavirus genomes circulating in bat reservoirs.

A continuation of sampling from Leschenault’s rousette and lesser dawn bat colonies in other geographic regions could reveal deeper insight into the circulation of RoBat-CoV GCCDC1 variants in wild bats. These bats inhabit regions between Singapore and Yúnnan province, China, and may also carry RoBat-CoV GCCDC1, based upon similarities in viruses detected among groups of the same species. For example, recent serological biosurveillance of lesser dawn bat populations in Singapore and Northeast India demonstrated that both populations had similar exposure to Asiatic filoviruses [45,46]. The geographic range of *E. spelaea* extends across Southeast Asia, and it is unknown whether these populations are panmictic. As RoBat-CoV GCCDC1 continues to circulate in co-roosting populations of multiple bat species, the spike gene will be under pressure, as demonstrated in Figure 2. While the cellular receptor for RoBat-CoV GCCDC1 and HKU9 is unknown, surveillance for adaptation and mutation of the spike gene should be performed, to estimate risk of tropism for receptors found in intermediate amplifying hosts or humans. 

Coronaviruses may emerge following random mutations permissive to infection of intermediate amplifying hosts, and/or recombination events that result in a large pool of variants that could infect humans, potentially without an intermediate host [24]. Due to the knowledge gap, with regard to the circulation of recombinant viruses, an understanding of the prevalence of unique, recombinant viruses will provide an advantage to predict the innate features of a virus with greater propensity for spillover. RoBat-CoV GCCDC1 is an intriguing, cross-family recombinant virus, with a geographical range that expands farther than was previously known. 

## Data Availability

The datasets supporting the conclusions of this article are available in National Center for Biotechnology Information (NCBI) Sequence Read Archive (SRA), BioProject ID PRJNA561193 and under GenBank accession number MT350598.

## Figures and Tables

**Table 1:** Summary of prevalence of RoBat-CoV GCCDC1 in swabs collected over the course of 18 months.

Sampling Date	# Swabs Sequenced	# Swabs Positive for GCCDC1 (%)	# Bats Sampled	# Bats Positive for GCCDC1 (%)
April 2016	41	11 (26.8%)	18	8 (44.4%)
July 2016	28	4 (14.3%)	19	4 (21.1%)
October 2016	75	55 (73.3%)	20	20 (100%)
January 2017	14	1 (7.1%)	11	1 (9.1%)
May 2017	21	0 (0%)	15	0 (0%)
September 2017	27	1 (3.7%)	13	1 (7.7%)

## Supplementary Materials

The following are available online at https://www.mdpi.com/1999-4915/12/5/539/s1, Table S1: Number of positive swabs detected at each time point, parsed by swab type.
