# Supplementary material for: Detection of Recombinant Rousettus Bat Coronavirus GCCDC1 in Lesser Dawn Bats (Eonycteris spelaea) in Singapore
Source: Viruses. 2020 May 14;12(5):539. doi: 10.3390/v12050539 (PMC7291116; doi:10.3390/v12050539)
Supplement: Supplementary file 1 [file viruses-12-00539-s001.pdf]

Supplemental Table 1. Number of positive swabs detected at each time point, parsed by swab type. A swab was considered to be positive if more than 10 reads mapped to more than 100 bases of the scaffolded reference genome with greater than 95% identity.

Date	Body swab	Head swab	Oral swab	Rectal swab
April 2016	2	3	3	3
July 2016			1	3
October 2016	18	18	6	13
January 2017	1			
September 2017			1	
